# Congenital Bilateral Multiple Trigger Fingers in A 5-Year-Old Child

**Published:** 2017-09

**Authors:** Neeraj Bhaban, Maksud Devale, Amarnath Munoli

**Affiliations:** Department of Plastic and Reconstructive Surgery, Mumbai, India

**Keywords:** Congenital, Trigger fingers, Child

## Abstract

Paediatric bilateral multiple trigger fingers are extremely rare. The underlying etiopathogenesis and hence the surgical principles of management of trigger finger in children are different from those of pediatric trigger thumb and adult trigger finger. In this paper, we report the case of a 5 year old girl with congenital trigger digits involving the middle, ring and little fingers of both hands. She did not have any episode of trauma, viral or bacterial infections or any metabolic disorder. Following lack of any improvement with a physiotherapy and a splintage regime for 6 weeks, we offered surgical management for the affected digits. Release was done in step-wise pattern. We present the intraoperative findings and surgical management of congenital trigger finger.

## INTRODUCTION

A trigger finger in the pediatric population is rare, affecting 0.005% of children,^1^ and multiple bilateral trigger digits in children are extremely rare.^2^ We present a case of bilateral multiple trigger fingers in a 5 year old female child.

## CASE REPORT

A 5-year-old girl was seen with complaints of inability to completely straighten the fingers of both hands. Initially the partly bent fingers could be straightened with “a pop” (triggering). Gradually a flexed posture of the Middle, Ring and Little fingers of both hands was noticed. There was no history of perinatal trauma, viral or bacterial infections, or rheumatologic or metabolic disorders, nor was there any other developmental anomaly.

The physical examination revealed flexion deformity ranging from 10-20 degrees in both proximal and distal interphalangeal joints of the middle, ring and little fingers of both hands. Triggering was absent. Non-tender nodules could be palpated on the volar aspect at the level of the proximal interphalangeal joints of all affected fingers. Radiographs of both hands were normal. A high frequency focused ultrasound of both hands showed mild thickening of A1 pulley in all fingers of the right hand and three fingers of the left hand. Following 6 weeks of physiotherapy with no discernible benefit, considering the age of the patient and the nonreducible nature of the deformity, we decided to proceed with surgical release.

**Fig. 1:**
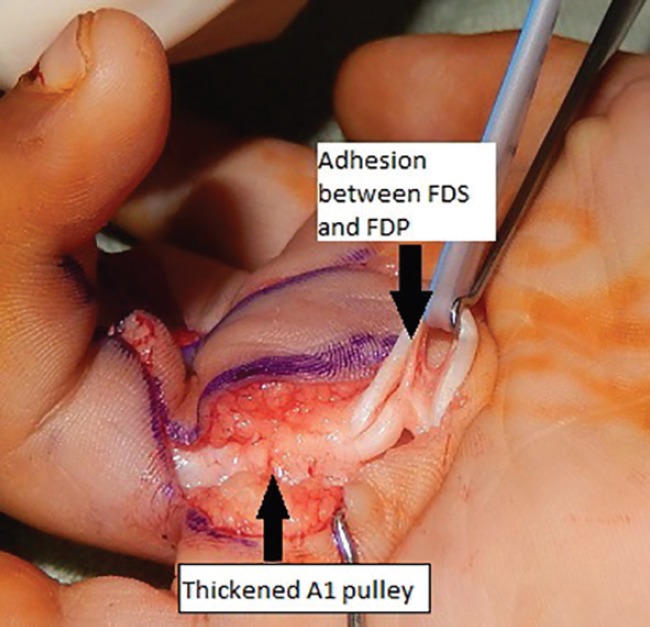
Arrow showing thickened A2 pulley and adhesion between FDS and FDP of ring finger.

**Fig. 2:**
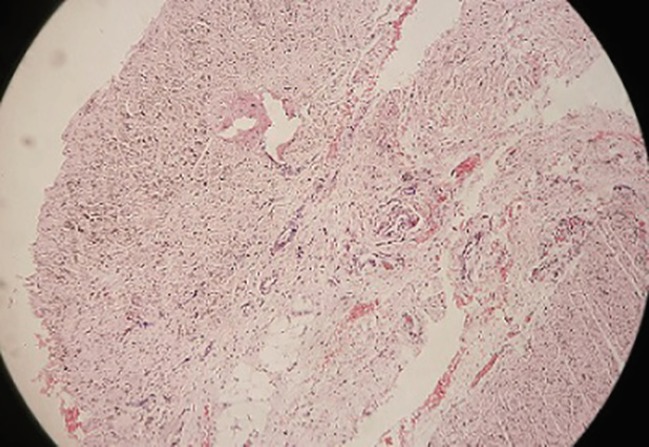
Microphotograph suggestive of mild inflammation at 100×.

Surgical exploration of the middle and ring fingers of the right hand was performed using Brunner’s incision. In both fingers considerable thickening of the A1 pulleys was noted. In the middle finger, fusiform enlargement of FDP tendon was found at the level of C1 pulley. In the ring finger, a fibrous band of adhesion was found between FDP and FDS tendons proximal to A1 pulley in addition to the above findings (Figure 1). After release of complete A1 pulley, partial A2 pulley and fibrous adhesion band, full passive extension was obtained in both middle and ring fingers. A part of the A1 pulley was sent for histopathological examination, which suggested mild inflammatory cellular infiltration (Figure 2). Postoperatively, range of motion exercises were started within pain limits from POD 2. Surgery for rest of the fingers is planned.

## DISCUSSION

Multiple aetiologies causing congenital trigger finger have been reported including anatomical variation, trauma, viral infection, inflammation,^1^ genetic disorders. Spontaneous resolution was reported in patients with mild symptoms or in young ages (24 months old or less).^1^ Most authors recommend surgical treatment, particularly if trigger finger persists after conservative treatment.^3^^-^^5^ Ultrasound scan has not proven a useful tool for predicting the site of triggering pre-operatively.^3^

Schaverien *et al.*^3^ reviewed literature and found that the predisposing anatomic problem can either be over crowding of the contents of the sheath (tendon nodules, wide flexor tendons, proximal or narrow decussation of FDS or aberrant connections between the flexor tendons) or a narrow pulley system (A1, A2 or A3). In our case, thickening A1 pulley was noted in both fingers. In addition, fusiform swelling of FDP of middle finger and adhesion between FDS and FDP of ring finger was found. Release of A1 pulley and partial A2 pulley lead to correction of deformity of both fingers. Thus surgical release of multiple trigger finger is different than in pediatric trigger thumb and adult trigger finger release. In pediatric trigger thumb and adult trigger finger release of A1 pulley is sufficient for correction of deformity. A more extensive surgical exposure is required in these cases to confirm release of all structures responsible for deformity. The operative algorithm given by Schaverien *et al.*^3^ suggests a step-wise approach to check all structures that may cause the symptom of triggering, whilst avoiding unnecessary dissection. With help of this algorithm we were able to achieve complete correction in this child.

## CONFLICT OF INTEREST

The authors declare no conflict of interest.
